# Activation of NLRP1 and NLRP3 inflammasomes contributed to cyclic stretch-induced pyroptosis and release of IL-1β in human periodontal ligament cells

**DOI:** 10.18632/oncotarget.11944

**Published:** 2016-09-10

**Authors:** Dan Zhao, Yaqin Wu, Jiabao Zhuang, Chun Xu, Fuqiang Zhang

**Affiliations:** ^1^ Department of Prosthodontics, Ninth People's Hospital, Shanghai Jiao Tong University School of Medicine, Shanghai, China; ^2^ Shanghai Key Laboratory of Stomatology & Shanghai Research Institute of Stomatology, Shanghai, China

**Keywords:** inflammasome, pyroptosis, IL-1β, cyclic stretch, human periodontal ligament cell

## Abstract

Inflammasomes have been reported to be present in periodontal inflammatory tissue, but the exact role of inflammasomes in periodontal inflammatory reactions especially those related to mechanical stimulations has not been clarified. In this study, it was shown that cyclic stretch activated the nucleotide-binding oligomerization domain-like receptor containing pyrin domain 1 and 3 (NLRP1 and NLRP3) inflammasomes and induced the release of IL-1β and pyroptosis via a caspase-1-related mechanism in human periodontal ligament cells (HPDLCs). This study firstly demonstrated that activation of NLRP inflammasomes contributed to the stretch-induced inflammatory response in HPDLCs. As inflammasomes have been reported to be involved in both programmed cell death and inflammation, further studies are required to elucidate the exact roles and signaling pathway of inflammasomes in stretch-induced periodontal inflammation.

## INTRODUCTION

Mechanical stress may affect the balance between tissue destruction and tissue regeneration. Under normal physiologic conditions, homeostasis of the periodontium is maintained by appropriate mechanical stimulation caused by occlusal load [1]. In contrast, abnormal mechanical stimulations aroused from occlusal overloading or improper orthodontic treatment may induce inflammatory response, which may subsequently lead to a pathological process in periodontium [2].

Previous studies reported that large numbers of inflammatory mediators, including pro-inflammatory cytokines (such as IL-1β, IL-6, IL-8 and tumor necrosis factor-alpha (TNF-α)), adenosine tri-phosphate (ATP), prostaglandin E_2_ (PGE_2_) and nitric oxide, were found in various cell types upon mechanical stimulation [3–5]. Among all these mediators, the release of IL-1β is considered significant in the initiation and regulation of inflammation [6]. Recently, a formation of a multisubunit complex named ‘inflammasome’ was reported, which consists of pro-inflammatory caspase(s), nucleotide-binding oligomerization domain-like receptor family members containing pyrin domain (NLRP), and the adaptor molecule apoptosis-associated speck-like protein containing a caspase-recruitment domain (ASC) [7, 8]. Among the known inflammasome complexes, NLRP1 and NLRP3 inflammasomes were found to function in generation of the inflammatory cytokine IL-1β [9, 10]. It was reported that mechanical stretch stimulated lung alveolar macrophages, which in turn activated caspase-1 in NLRP3 inflammasome, leading to the activation and release of IL-1β [11].

Inflammatory reactions in periodontal tissue in response to mechanical stress have also been reported [12]. It was reported that some cytokines, such as IL-1β, IL-6, IL-8, TNF-α and interferon-γ, were induced in response to mechanical stress in animal and human periodontal tissues [13–15]. We have previously reported that mechanical stretch induced programmed cell death and the increased gene expression of inflammatory caspase in HPDLCs [16–18].

NLRP inflammasomes were concerned with pyroptosis, which is a recently described pathway of programmed cell death. Unlike apoptosis which is silent with respect to inflammation, pyroptosis is characterized by release of danger signals and subsequent induction of inflammatory immune responses [19]. NLRP inflammasomes have also been reported to be present in periodontal inflammatory tissue [20], but the exact role of inflammasomes in periodontal inflammation has not been clarified. As mechanical stimulations including stretching have been proved to be able to provoke inflammatory reaction in periodontal tissue and programmed cell death, whether NLRP inflammasomes were expressed and activated in HPDLCs in response to stretch, and whether their expression and activation contributed to the stretch-related periodontal inflammation become interesting issues and need to be elucidated. Thus, in the present study, we investigated the role of NLRP inflammasomes in the inflammatory reaction of HPDLCs stimulated by *in vitro* stretch.

## RESULTS

### NLRP1 and NLRP3 expressed in HPDLCs in response to cyclic stretch

**Figure 1:**
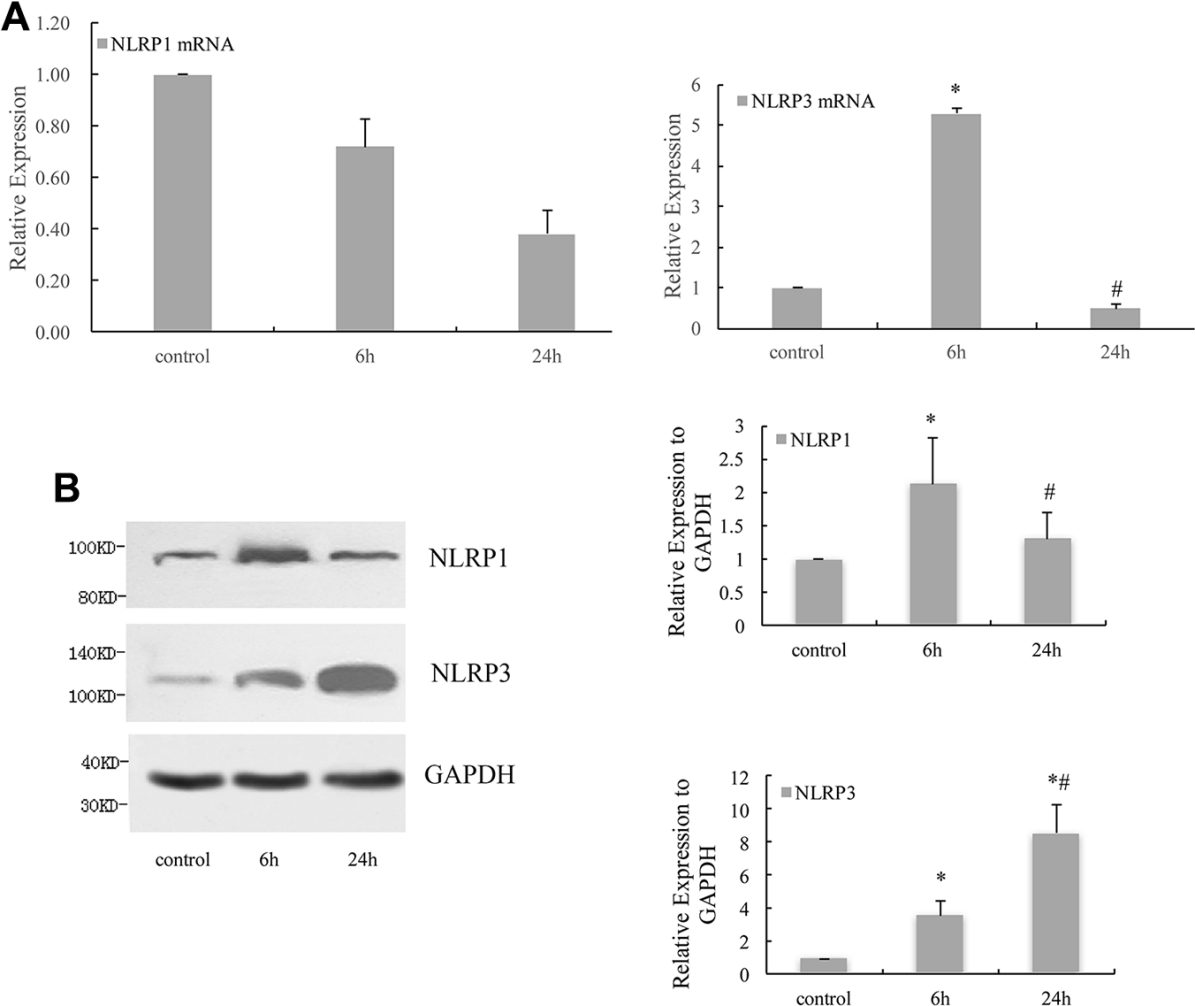
NLRP1 and NLRP3 expressed in HPDLCs in response to cyclic stretch (**A**) Real-time PCR result of NLRP1 in HPDLCs showed no significant difference among different groups, while the results of NLRP3 showed that its mRNA increased in response to 6 h cyclic stretch significantly and decreased to the control level after 24 h stretch. (**B**) Western blot analysis revealed that the expressions of NLRP1 and NLRP3 increased in response to 6 h cyclic stretch. Expression of NLRP3 further increased in response to 24 h cyclic stretch. The expression of NLRP1 decreased to the control level after 24 h cyclic stretch. GAPDH was used as a loading control. The results were quantified from at least 3 independent experiments and expressed as mean ± S.E. Statistical significance was calculated using one-way ANOVA with multiple comparisons. *P < 0.05 versus control group; ^#^*P* < 0.05 versus 6 h group.

Real-time PCR result showed that the expression of NLRP3 mRNA in HPDLCs increased significantly in response to 6 h cyclic stretch (*P* < 0.05 VS control), while the expression of NLRP1 mRNA did not change significantly. (Figure 1A) The analysis of Western blot showed that 6 h cyclic stretch increased the expressions of NLRP1 and NLRP3 (*P* < 0.05 VS control). Expression of NLRP3 further increased in response to 24 h cyclic stretch (*P* < 0.05 VS control and 6 h). While the expression of NLRP1 decreased to the control level after 24 h cyclic stretch (*P* > 0.05 VS control, *P* < 0.05 VS 6 h). (Figure 1B).

### Cyclic stretch induced the activation and release of IL-1β in HPDLCs

**Figure 2:**
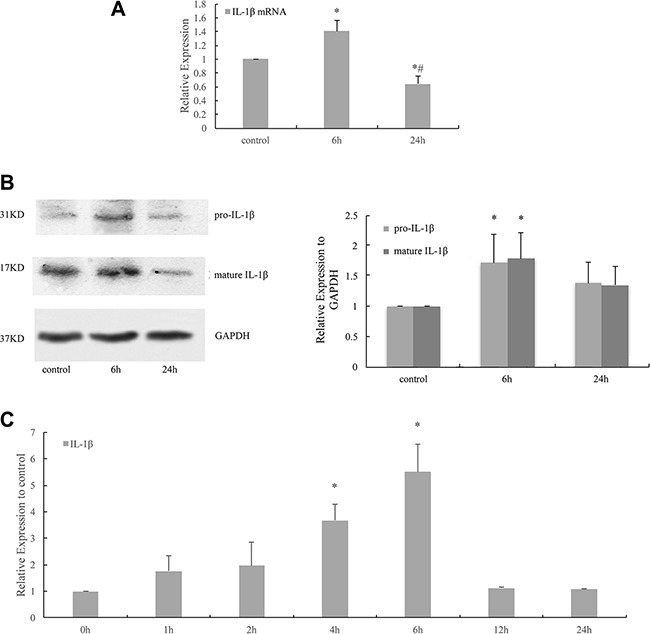
Cyclic stretch induced the activation and release of IL-1β in HPDLCs (**A**) Real-time PCR result revealed that the expression of IL-1β mRNA increased in response to 6 h cyclic stretch and was reduced after 24 h cyclic stretch. (**B**) Western blot analyses showed that 6 h cyclic stretch increased the expressions of pro-IL-1β and mature IL-1β proteins. GAPDH was used as a loading control. (**C**) ELISA assay verified the appearance of IL-1β in the cell-culture medium of HPDLCs in response to 1, 2, 4, 6, 12 and 24 h cyclic stretches and showed that both 4 and 6 h stretches stimulated the release of IL-1β. The appearance of IL-1β in the cell-culture medium of HPDLCs reached a peak after 6 h stretch and then decreased to the control level after 12 h stretch. The results were quantified from at least 3 independent experiments and expressed as mean ± S.E. Statistical significance was calculated using one-way ANOVA with multiple comparisons. *P < 0.05 versus control group; ^#^*P*< 0.05 versus 6 h group.

Real-time PCR result revealed that the expression of IL-1β mRNA increased in response to 6 h cyclic stretch (*P* < 0.05 VS control) and was reduced after 24 h cyclic stretch (*P* < 0.05 VS control, *P* < 0.05 VS 6 h). (Figure 2A) Western blot analysis showed that 6 h cyclic stretch increased the expressions of pro-IL-1β and mature IL-1β proteins (*P* < 0.05 VS control). (Figure 2B) The appearance of IL-1β in the cell-culture medium of HPDLCs in response to 1, 2, 4, 6, 12 and 24 h cyclic stretches were verified by ELISA assay. The result of ELISA showed that both 4 and 6 h stretches increased the release of IL-1β (*P* < 0.05 VS control). The appearance of IL-1β in the cell-culture medium of PDLCs reached a peak after 6 h stretch and then decreased to the control level after 12 h stretch. (Figure 2C).

### Cyclic stretch activated caspase-1 and -5 in HPDLCs

**Figure 3:**
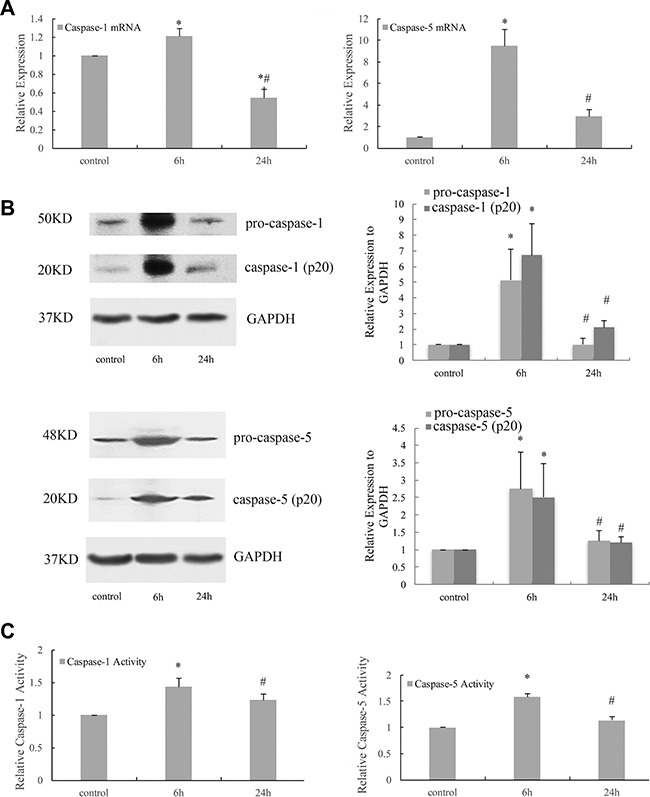
Cyclic stretch activated caspase-1 and -5 in HPDLCs (**A**) Real-time PCR result showed that the expressions of caspase-1 and -5 mRNA in human PDLCs increased in response to 6 h cyclic stretch and decreased after 24 h cyclic stretch. (**B**) Western blot analysis showed that 6 h cyclic stretch increased the expressions of pro-caspase-1, caspase-1 (p20), pro-caspase-5 and caspase-5 (p20). The expressions decreased to the control level after 24 h cyclic stretch. GAPDH was used as a loading control. (**C**) The activities of caspase-1 and -5 increased substantially after 6 h cyclic stretch. The results were quantified from at least 3 independent experiments and expressed as mean ± S.E. Statistical significance was calculated using one-way ANOVA with multiple comparisons. *P < 0.05 versus control group; ^#^*P* < 0.05 versus 6 h group.

Real-time PCR result showed that the expressions of caspase-1 and -5 mRNA in HPDLCs increased in response to 6 h cyclic stretch (*P* < 0.05 VS control). (Figure 3A) The analysis of Western blot showed that 6 h cyclic stretch increased the expressions of pro-caspase-1, caspase-1 (p20), pro-caspase-5 and caspase-5 (p20) (*P* < 0.05 VS control). And the expressions decreased to the control level after 24 h cyclic stretch (*P* > 0.05 VS control, *P* < 0.05 VS 6 h). (Figure 3B) The activities of caspase-1 and -5 increased substantially after 6 h cyclic stretch (*P* < 0.05 VS control). (Figure 3C).

### Cyclic stretch promoted the formation and activation of inflammasome in HPDLCs

**Figure 4:**
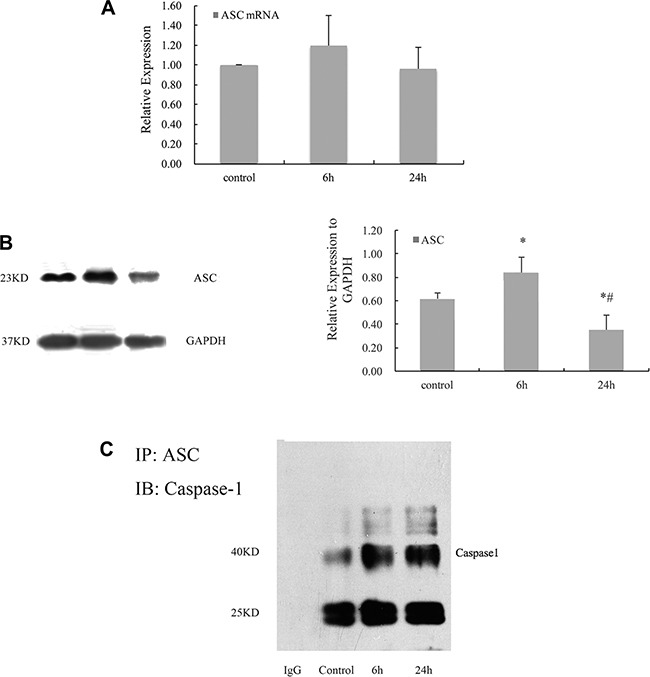
Cyclic stretch promoted the formation and activation of inflammasome in HPDLCs (**A**) Real-time PCR result showed that ASC mRNA did not change significantly in response to cyclic stretch. (**B**) Western blot analysis showed that 6 h cyclic stretch increased the expression of ASC, and 24 h cyclic stretch reduced its expression. GAPDH was used as a loading control. (**C**) The assembly of inflammasome was detected using immunoprecipitation (IP) with anti-ASC Ab followed by immunoblotting (IB) for caspase-1. The results were quantified from at least 3 independent experiments and expressed as mean ± S.E. Statistical significance was calculated using one-way ANOVA with multiple comparisons. *P < 0.05 versus control group; ^#^*P* < 0.05 versus 6 h group.

The expression of ASC mRNA did not change significantly in response to 6 and 24 h cyclic stretch. While the analysis of Western blot showed that 6 h cyclic stretch increased the expression of ASC (*P* < 0.05 VS control). (Figure 4A, 4B) Co-immunoprecipitation revealed that the expression of caspase-1 in cyclic stretched HPDLCs was immunoprecipitated with ASC, the adaptor protein in most inflammasomes. (Figure 4C).

### Role of caspase-1 in cyclic stretch-induced expression of IL-1β and pyroptosis

**Figure 5:**
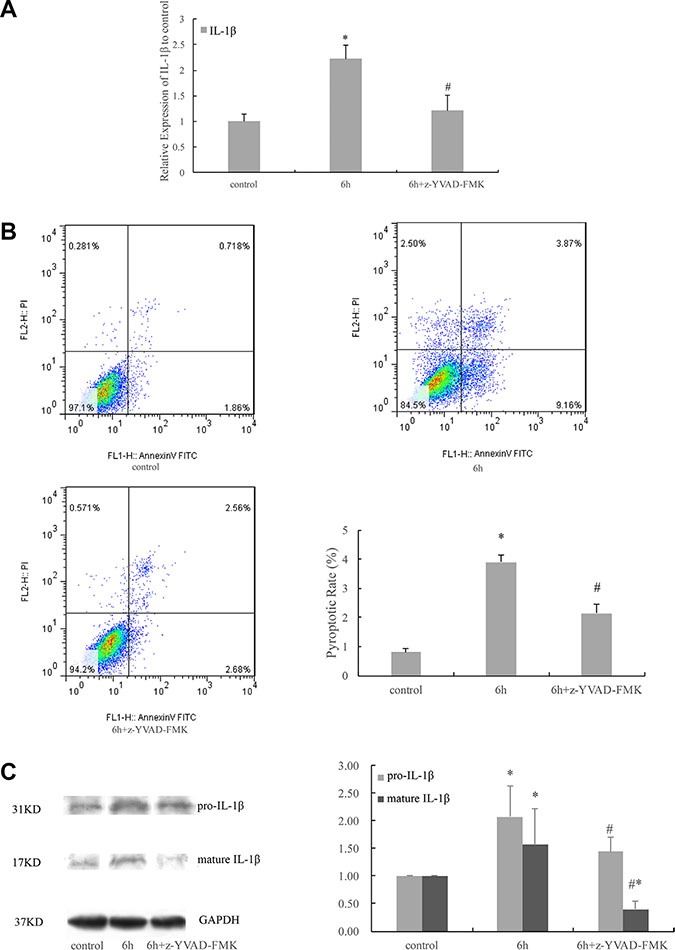
Cyclic stretch-induced expression of IL-1β and pyroptosis were caspase-1 dependent (**A**) The addition of the caspase-1 inhibitor significantly inhibited the appearance of IL-1β in the culture medium of the cyclic stretched cells, compared with the non-inhibited 6 h stretched cells. (**B**) The pyroptotic rate of HPDLCs in response to 6 h cyclic stretch increased significantly. While the addition of the caspase-1 inhibitor significantly inhibited the pyroptotic rate in response to 6 h cyclic stretch, compared with the non-inhibited 6 h stretched cells. (**C**) Western blot analysis showed that 6 h cyclic stretch increased the expressions of pro-IL-1β and mature IL-1β proteins. While the addition of the caspase-1 inhibitor significantly inhibited the expressions of both pro-IL-1β and mature IL-1β, compared with the non-inhibited 6 h stretched cells. The GAPDH was used as a loading control. results were quantified from at least 3 independent experiments and expressed as mean ± S.E. Statistical significance was calculated using one-way ANOVA with multiple comparisons. *P < 0.05 versus control group; ^#^*P* < 0.05 versus 6 h group.

Compared with the non-stretching control, the appearance of IL-1β in the culture medium of HPDLCs increased significantly in response to 6 h cyclic stretch (*P* < 0.05 VS control). While the addition of the caspase-1 inhibitor, z-YVAD-FMK, significantly inhibited the appearance of IL-1β in the culture medium of the cyclic stretched cells, compared with the non-inhibited 6 h stretched cells (*P* < 0.05 VS 6 h). (Figure 5A) Flow cytometric analysis of cells double-labelled with annexin V-FITC and PI to differentiate healthy (FITC −/PI −), apoptotic (FITC +/PI −) and necrotic/pyroptotic (FITC +/PI +) cells, showed that the pyroptotic rate of HPDLCs in response to 6 h cyclic stretch increased significantly (*P* < 0.05 VS control). While the addition of the caspase-1 inhibitor significantly inhibited the pyroptotic rate in response to 6 h cyclic stretch, compared with the non-inhibited 6 h stretched cells (*P* < 0.05 VS 6 h) (Figure 5B). Western blot analysis showed that 6 h cyclic stretch increased the expressions of pro-IL-1β and mature IL-1β proteins (*P* < 0.05 VS control). While the addition of the caspase-1 inhibitor significantly inhibited the expressions of both pro-IL-1β and mature IL-1β, compared with the non-inhibited 6 h stretched cells (*P* < 0.05 VS 6 h). (Figure 5C).

## DISCUSSION

To our knowledge, this is the first study to probe the involvement of NLRP inflammasome signaling in periodontal inflammation provoked by mechanical stretch. We demonstrated that the expressions of IL-1β, caspase-1, caspase-5, ASC, NLRP1, and NLRP3 were induced in HPDLCs by mechanical stretch loading.

In the present study, the expressions of IL-1β mRNA and protein in HPDLCs, as well as the secreted IL-1β all remarkably increased in response to stretch. This was in agreement with previous reports that HPDLCs released pro-inflammatory cytokines, including TNF-α, receptor activator of nuclear factor-kappaB ligand (RANKL), matrix metalloproteinase (MMP), cyclooxygenase-2 (COX-2) and IL-6, during orthodontic tooth movement [13, 21–23]. Moreover, high level of IL-1 was also found in HPDLCs in response to mechanical stress both *in* vivo and *in vitro* [12, 14, 24]. It was shown that the IL-1β in HPDLCs, through stimulation of COX-2 expression and PGE2 synthesis, activated IL-8 and promoted the expression of MMPs, so as to promote the damage of periodontal soft tissue and the absorption of periodontal bone [13, 15, 23]. Taken together, it was speculated that periodontal inflammation might be induced by mechanical stretch with IL-1β activation as the pivot in this process.

**Figure 6:**
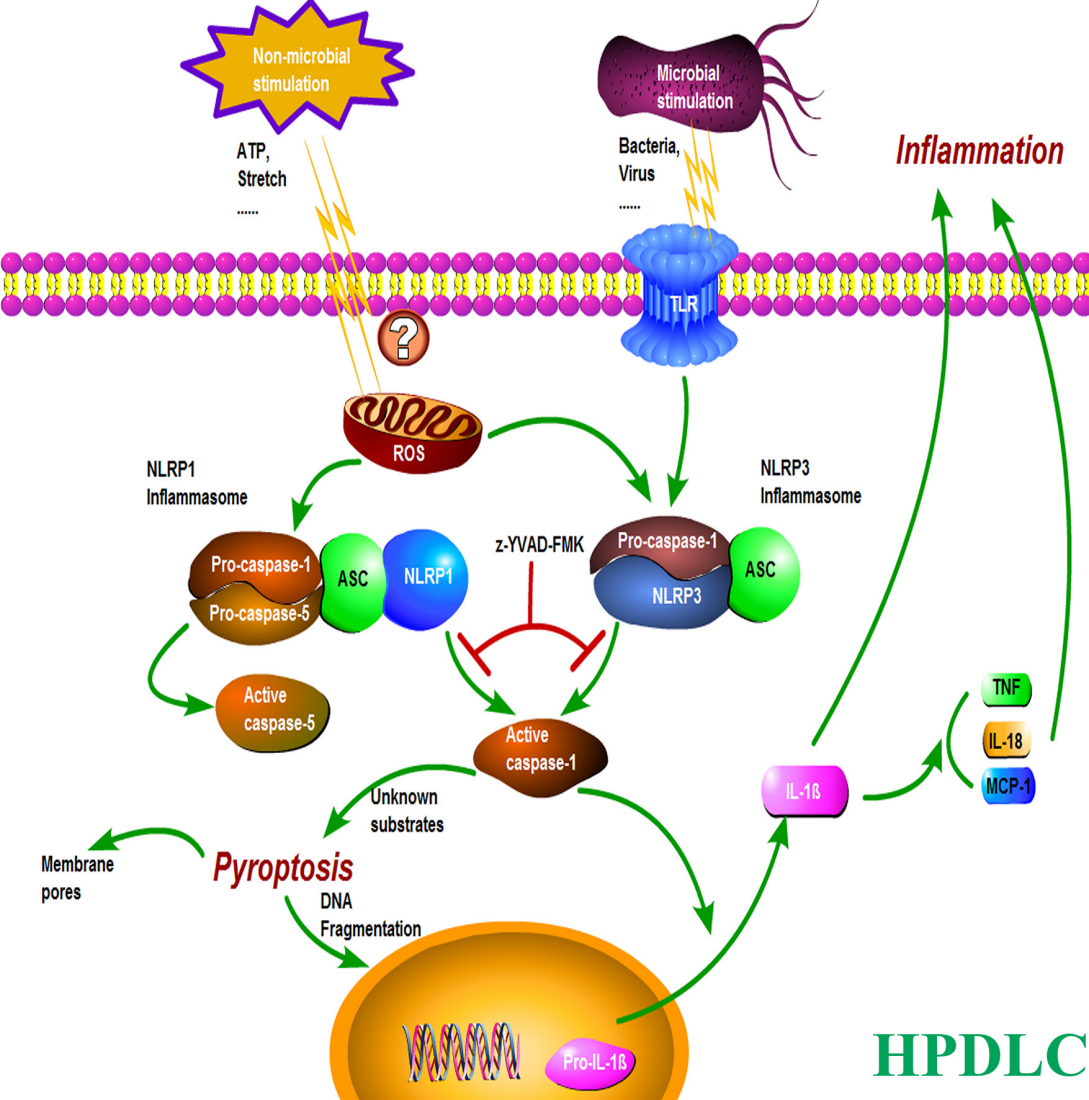
A proposed model illustrating the mechanism of inflammation and pyroptosis via NLRP3 and NLRP1 inflammasomes in HPDLCs.

Inflammasomes are large cytosolic multiprotein complexes that assemble in response to detection of infection- or stress-associated stimuli and lead to the caspase-1-mediated inflammatory responses, including activation of pro-inflammatory cytokines IL-1β and IL-18, and initiation of an inflammatory form of cell death referred to as pyroptosis, which features pore formation in the plasma membrance [25]. (Figure 6) To date the function of most NLRs remains elusive, among which NLRP1 and NLRP3 are the most extensively studied and are known to form inflammasomes that activate caspase-1 [26].

NLRP3 has become the focus of increasing attention in the research of inflammation and is by far the most thoroughly studied NLRs [27]. Recently it was reported that mechanical ventilation resulted in inflammation associated with lung injury via NLRP3 inflammasome [28]. NLRP3 inflammasome has also been reported to express in periodontal diseases [20]. However, inflammasome activation in HPDLCs has not been well studied. The caspase-1-related activation of IL-1β and pyroptosis, and the elevated expressions of NLRP3 and activated caspase-1 induced by stretch, together with the immunoprecipitation reaction between caspase-1 and ASC found in the present study, highly suggested that NLRP3 inflammasome was formed and activated in human PDLCs by cyclic stretching. The NLRP3 inflammasome cleaved the pro-caspase-1 to active caspase-1, which then leaded to processing and maturation of the pro-inflammatory cytokine IL-1β and initiation of the inflammatory cell death pyroptosis. (Figure 6) By far three models for NLRP3 inflammasome activation have been proposed: (1) the ion flux model, (2) the reactive oxygen species (ROS) model, and (3) the lysosome rupture model. Despite considerable efforts, there is currently no agreement on an universal mechanism by which the NLRP3 inflammasome is activated [25]. So further studies on the mechanism of the stretch-induced activation of the NLRP3 inflammasome signaling pathway should be conducted in PDLCs.

NLRP1 inflammasome, as an important danger-sensing platform, has been found to express in many tissues. Previous studies showed that NLRP1 inflammasome functioned in regulation of inflammatory caspases and consequent processing of IL-1β [29]. This inflammasome is comprised of caspase-1, caspase-5, ASC and NLRP1. In the present study, in addition to the stretch-induced expressions of caspase-1 and ASC, increased expression of NLRP1 and activation of caspase-5 in response to stretch was also found in HPDLCs. This suggested that NLRP1 inflammasome was also formed and activated in HPDLCs by cyclic stretching. The NLRP1 inflammasome led to the activation of caspase-1 and the subsequent activation and releasing of IL-1β, and initiated pyroptosis. (Figure 6) Likely the mechanism of human NLRP1 inflammasome activation was different from NLRP3. It was observed that the activation of NLRP1 inflammasome was concerned with synthetic muramyl dipeptide (MDP) [29]. The exact mechanism of the stretch-induced activation of the NLRP1 inflammasome signaling pathway should be further studied in HPDLCs.

Our study is limited in that only one downstream NLRP target (IL-1β) was investigated. IL-1β is considered significant in the initiation and regulation of inflammation, so the detection of IL-1β was selected. Another limitation is that only the inhibitor of caspase-1 was used in the inhibitory experiment. As proteolytic processing for caspase-1 is the hallmark of NLRP inflammasome activation [30], the specific role of caspase-1 was detected in the present study.

In summary, to our knowledge, this is the first study demonstrated that NLRP inflammasomes were activated and contributed to the stretch-induced inflammatory response in HPDLCs. Inflammation in the periodontal ligament may contribute to the resorption of alveolar bone, possibly facilitate the development of apical periodontitis. And periodontal inflammation has also been suggested in the recent years as a potential factor influencing the development of many system diseases [31, 32]. As inflammasomes have been reported to be involved in both programmed cell death and inflammation, further studies are required to elucidate the exact roles and signaling pathway of inflammasomes in stretch-induced periodontal inflammation and programmed cell death.

## MATERIALS AND METHODS

### Cell cultures

HPDLCs were obtained from healthy premolars extracted from children aged 11–13 year for orthodontic reasons, after informed consents were obtained from their parents. The protocol for harvesting human periodontal tissue from extracted teeth was approved by the Ethics Committee of Ninth People's Hospital, Shanghai Jiao Tong University School of Medicine (Reference: [2008]17). HPDLCs were cultured as previously described [33]. HPDL on the middle of the root was acquired with a sterile scalpel. Then pieces of HPDL were attached to a cell culture dish and cultured in Dulbecco's modification of Eagle's medium (DMEM, Gibco, Grand Island, NY, USA) supplemented with 20% (v/v) fetal bovine serum (FBS, Hyclone, Logan, UT, USA) and five-fold reinforced antibiotics (500 U/mL penicillin and 500 μg/mL streptomycin, Sigma-Aldrich, St. Louis, MO, USA) at 37°C in a humidified atmosphere of air containing 5% CO_2_. Cells that grew out from the extracts were passaged in DMEM supplemented with 10% (v/v) FBS and antibiotics (100 U/mL penicillin and 100 μg/mL streptomycin). Cells at passage 4–6 were used in the present study.

### Stretch loading

For cyclic stretch loading experiments, HPDLCs were seeded onto six-well Bioflex plates (Flexcell International, Hillsborough, NC, USA) at a density of 3 × 10^5^ cells/well. Cells reached confluence following 3 d of culture and then were subjected to cyclic stretch using a Flexcell Tension Plus system (FX-5000T, Flexcell International) with a 20% elongation magnitude for 6 or 24 h. Cells cultured on the same kind of plates but without stretch loading were used as time-matched control cells. The loading frequency of 6 cycles/min (5 s stretch and 5 s relaxation) was the same to that in our previous studies [17, 18]. Three independent loading experiments were carried out in every group.

### RNA isolation and quantitative real-time PCR

After the application of cyclic stretch, total RNA was extracted from HPDLCs using Trizol reagent (Life Technologies), according to manufacturer's recommendations. cDNA was synthesized using a Reverse Transcription Kit (Toyobo, Tokyo, Japan), according to the manufacturer's instructions. Real-time PCR was carried out using an ABI 7500 Real-Time PCR System (Applied Biosystems, Sunnyvale, CA, USA). To control variability in amplification due to differences in starting mRNA concentrations, β-actin was used as an internal control. The designs of PCR primers are shown in Table 1.

**Table 1:** PCR primers

Gene	Sequence
GAPDH	F:5′ GGGAAACTGTGGCGTGAT 3′ R:5′ GAGTGGGTGTCGCTGTTGA 3′
CASP1	F:5′ GGACAAGTCAAGCCGCACA 3′ R:5′ CATGTCCGAAGCAGTGAGAT 3′
CASP5	F:5′ AGATATTCAACAACCGCAACTG 3′ R:5′ AAGAGATGAGTGCCAAGGATG 3′
NLRP1	F:5′ CCCCTCTATCGGCGTCTATCT 3′ R:5′ CCTTTGCCTTGGCTCTTACC 3′
NLRP3	F:5′ GAGCACCAGCCAGAGTCTAA 3′ R:5′ CCGAATGTTACAGCCAGGATGC 3′
IL-1β	F:5′ CGAATCTCCGACCACCACTACA 3′ R:5′ AGGGAACCAGCATCTTCCTCAG 3′

### Western blot analysis

After the application of cyclic stretch, HPDLCs were washed with ice-cold PBS, scraped from the Bioflex plates and immediately lysed in a lysis buffer (Cell Signaling Technology, Danvers, MA, USA). Protein concentrations of the samples were determined by using the bicinchoninic acid protein assay kit (Pierce, Rockford, IL, USA). Protein was mixed with an appropriate volume of SDS sampling buffer and separated by SDS-PAGE gel (10%). The protein bands were then transferred onto nitrocellulose membranes by electroblotting. According to the manufacturer's instructions, the membranes were incubated with mouse monoclonal antibody against caspase-1(1:1000, Cell Signaling Technology), caspase-5 (1:1000, Cell Signaling Technology), ASC (1:1000, Santa Cruz Biotechnology, Dallas, TX, USA), NLRP1(1:1000, Abcam), NLRP3(1:1000, Abcam), GAPDH (1:10000, Cell Signaling Technology), or rabbit anti-IL-1β (1:1000, Cell Signaling Technology) primary antibodies overnight at 4°C, washed, and then incubated with anti-mouse or anti-rabbit IgG conjugated to HRP (1:5000, Cell Signaling Technology) for 60 min at room temperature. Protein bands were detected by using the ECL SuperSignal reagent (Pierce). Relative band densities of the proteins were measured from scanned films using National Institutes of Health ImageJ Software.

### Cytokine elisa

After the application of cyclic stretch, the protein concentration of IL-1β in the culture supernatants from cultured HPDLCs were estimated using an IL-1β ELISA kit (R&D Systems, Minneapolis, MN, USA), according to the manufacturer's instruction. 150 μL cell-culture medium was collected from each sample and added into well of the microplate, and then the microplate was washed with Wash Buffer. Each well was incubated with IL-1β high sensitivity Conjugate for 2 h at room temperature on the shaker and then was washed with Wash Buffer. 50 μL of Substrate Solution was added into each well, and incubated for 1 h at room temperature on the shaker. After Stop Solution was added, the optical density of each well was determined using a microplate reader (Elx800, Biotec, Vermont, USA) set to 490 nm.

### Caspase-1, -5 activity

Caspases' activities were measured using the corresponding caspase colorimetric assay kit (Biovision Research Products, Mountain View, CA, USA), according to the manufacturer's instruction. After the application of cyclic stretch, the cells were washed with PBS and suspended in 50 ul of chilled cell lysis buffer for 10 min on ice. The cell suspension was homogenized by trituration with a syringe before being centrifuged for 1 min in a microcentrifuge (10,000 g). Supernatant was harvested and transferred to a fresh tube and kept on ice for immediate assay. The protein content of the supernatant was quantified with the Micro BCA™ protein assay reagent kit (Pierce). Protein (100 ug) diluted in 50 ul cell lysis buffer was added to 50 ul of reaction buffer containing 10 mM DL-Dithiothreito (DTT), and then 5 ul of 4 mM p-nitroanilide (pNA) (YVAD-pNA for caspase-1, WEHD-pNA for caspase-5) was added into the buffer. The mixture was incubated at 37°C for 1 h. The absorbance at 405 nm was measured using a microplate reader (Elx800, Biotec).

### Co-immunoprecipitation

50 μl of protein concentrations of the samples was incubated with 1 μl anti-ASC antibody (sc-22514-R, Santa Cruz, CA, USA) overnight at 4°C. This reaction mixture was then incubated with protein A magnetic beads (sc-2003, Santa Cruz) for 30 min at 4°C. Precipitates were washed three times with washing buffer and then eluted from protein A magnetic beads by boiling with 1 × SDS for 10 min at 90–100°C. Western blot analysis was used to evaluate the expression of caspase-1 with an anti-caspase-1 antibody (1:1000, Cell Signaling Technology). Homophytic IgG was used as the negative control in Western blot analysis.

### Inhibitor treatment

For inhibitory studies, HPDLCs were pre-incubated with 2 mM caspase-1 inhibitor (z-YVAD-FMK) (Biovision Research Products) for 1 h before cyclic stretch loading. HPDLCs under the same conditions but without inhibitor were used as non-inhibiting controls.

### Annexin V-FITC and Propidium Iodide (PI) labeling

HPDLCs exposed to 20% cyclic stretch strain for 6 h were washed twice with PBS, and phosphatidylserine exposure and loss of membrane integrity was assessed simultaneously by labeling with FITC-coupled annexin V and propidium iodide (PI) (BD Biosciences, San Jose, CA, USA). Labeling was quantified by flow cytometry using a FACStar Plus flow cytometer (BD Pharmingen, Oakville, ON, USA).

### Data analysis

The results were presented as mean ± standard deviation (*n* = 3) and statistically analyzed with one-way analysis of variance (ANOVA) followed by the least-significant difference (LSD) test or the *t* test for paired samples. *P* values of less than 0.05 were considered statistically significant.

## References

[1] Davidovitch Z, Nicolay OF, Ngan PW, Shanfeld JL (1988). Neurotransmitters, cytokines, and the control of alveolar bone remodeling in orthodontics. Dent Clin North Am.

[2] Kaku M, Uoshima K, Yamashita Y, Miura H (2005). Investigation of periodontal ligament reaction upon excessive occlusal load—osteopontin induction among periodontal ligament cells. J Periodontal Res.

[3] Chowdhury B, David AL, Thrasivoulou C, Becker DL, Bader DL, Chowdhury TT (2014). Tensile strain increased COX-2 expression and PGE2 release leading to weakening of the human amniotic membrane. Placenta.

[4] Karadottir H, Kulkarni NN, Gudjonsson T, Karason S, Gudmundsson GH (2015). Cyclic mechanical stretch down-regulates cathelicidin antimicrobial peptide expression and activates a pro-inflammatory response in human bronchial epithelial cells. PeerJ.

[5] Lin YM, Li F, Shi XZ (2014). Mechanical stress is a pro-inflammatory stimulus in the gut: *in vitro*, *in vivo* and *ex vivo* evidence. PLoS One.

[6] Martinon F, Burns K, Tschopp J (2002). The inflammasome: a molecular platform triggering activation of inflammatory caspases and processing of proIL-beta. Mol Cell.

[7] Schroder K, Tschopp J (2010). The inflammasomes. Cell.

[8] van de Veerdonk FL, Netea MG, Dinarello CA, Joosten LA (2011). Inflammasome activation and IL-1beta and IL-18 processing during infection. Trends Immunol.

[9] Ganter MT, Roux J, Miyazawa B, Howard M, Frank JA, Su G, Sheppard D, Violette SM, Weinreb PH, Horan GS, Matthay MA, Pittet JF (2008). Interleukin-1beta causes acute lung injury via alphavbeta5 and alphavbeta6 integrin-dependent mechanisms. Circ Res.

[10] Martinon F, Mayor A, Tschopp J (2009). The inflammasomes: guardians of the body. Annu Rev Immunol.

[11] Wu J, Yan Z, Schwartz DE, Yu J, Malik AB, Hu G (2013). Activation of NLRP3 inflammasome in alveolar macrophages contributes to mechanical stretch-induced lung inflammation and injury. J Immunol.

[12] Ren Y, Vissink A (2008). Cytokines in crevicular fluid and orthodontic tooth movement. Eur J Oral Sci.

[13] Jacobs C, Walter C, Ziebart T, Grimm S, Meila D, Krieger E, Wehrbein H (2014). Induction of IL-6 and MMP-8 in human periodontal fibroblasts by static tensile strain. Clin Oral Investig.

[14] Bletsa A, Berggreen E, Brudvik P (2006). Interleukin-1alpha and tumor necrosis factor-alpha expression during the early phases of orthodontic tooth movement in rats. Eur J Oral Sci.

[15] Maeda A, Soejima K, Bandow K, Kuroe K, Kakimoto K, Miyawaki S, Okamoto A, Matsuguchi T (2007). Force-induced IL-8 from periodontal ligament cells requires IL-1beta. J Dent Res.

[16] Xu C, Hao Y, Wei B, Ma J, Li J, Huang Q, Zhang F (2011). Apoptotic gene expression by human periodontal ligament cells following cyclic stretch. J Periodontal Res.

[17] Zhong W, Xu C, Zhang F, Jiang X, Zhang X, Ye D (2008). Cyclic stretching force-induced early apoptosis in human periodontal ligament cells. Oral Dis.

[18] Hao Y, Xu C, Sun SY, Zhang FQ (2009). Cyclic stretching force induces apoptosis in human periodontal ligament cells via caspase-9. Arch Oral Biol.

[19] Fink SL, Cookson BT (2006). Caspase-1-dependent pore formation during pyroptosis leads to osmotic lysis of infected host macrophages. Cellular microbiology.

[20] Bostanci N, Emingil G, Saygan B, Turkoglu O, Atilla G, Curtis MA, Belibasakis GN (2009). Expression and regulation of the NALP3 inflammasome complex in periodontal diseases. Clin Exp Immunol.

[21] Shimizu N, Yamaguchi M, Goseki T, Ozawa Y, Saito K, Takiguchi H, Iwasawa T, Abiko Y (1994). Cyclic-tension force stimulates interleukin-1 beta production by human periodontal ligament cells. J Periodontal Res.

[22] Ritter N, Mussig E, Steinberg T, Kohl A, Komposch G, Tomakidi P (2007). Elevated expression of genes assigned to NF-kappaB and apoptotic pathways in human periodontal ligament fibroblasts following mechanical stretch. Cell Tissue Res.

[23] Shimizu N, Ozawa Y, Yamaguchi M, Goseki T, Ohzeki K, Abiko Y (1998). Induction of COX-2 expression by mechanical tension force in human periodontal ligament cells. J Periodontol.

[24] Uematsu S, Mogi M, Deguchi T (1996). Interleukin (IL)-1 beta, IL-6, tumor necrosis factor-alpha, epidermal growth factor, and beta 2-microglobulin levels are elevated in gingival crevicular fluid during human orthodontic tooth movement. J Dent Res.

[25] de Zoete MR, Palm NW, Zhu S, Flavell RA (2014). Inflammasomes. Cold Spring Harb Perspect Biol.

[26] Aachoui Y, Sagulenko V, Miao EA, Stacey KJ (2013). Inflammasome-mediated pyroptotic and apoptotic cell death, and defense against infection. Curr Opin Microbiol.

[27] Zhao J, Wang H, Dai C, Wang H, Zhang H, Huang Y, Wang S, Gaskin F, Yang N, Fu SM (2013). P2X7 blockade attenuates murine lupus nephritis by inhibiting activation of the NLRP3/ASC/caspase 1 pathway. Arthritis Rheum.

[28] Kuipers MT, Aslami H, Janczy JR, van der Sluijs KF, Vlaar AP, Wolthuis EK, Choi G, Roelofs JJ, Flavell RA, Sutterwala FS, Bresser P, Leemans JC, van der Poll T (2012). Ventilator-induced lung injury is mediated by the NLRP3 inflammasome. Anesthesiology.

[29] Chavarria-Smith J, Vance RE (2015). The NLRP1 inflammasomes. Immunol Rev.

[30] Jin C, Flavell RA (2010). Molecular mechanism of NLRP3 inflammasome activation. J Clin Immunol.

[31] Nguyen CM, Kim JW, Quan VH, Nguyen BH, Tran SD (2015). Periodontal associations in cardiovascular diseases: The latest evidence and understanding. J Oral Biol Craniofac Res.

[32] Sete MR, Figueredo CM, Sztajnbok F (2016). [Periodontitis and systemic lupus erythematosus] [Article in English, Portuguese]. Rev Bras Reumatol.

[33] Xu C, Fan Z, Shan W, Hao Y, Ma J, Huang Q, Zhang F (2012). Cyclic stretch influenced expression of membrane connexin 43 in human periodontal ligament cell. Arch Oral Biol.

